# Tumor-Associated Macrophages: New Horizons for Pituitary Adenoma Researches

**DOI:** 10.3389/fendo.2021.785050

**Published:** 2021-12-02

**Authors:** Changxi Han, Shaojian Lin, Xingyu Lu, Li Xue, Zhe Bao Wu

**Affiliations:** Department of Neurosurgery, Center of Pituitary Tumor, Ruijin Hospital, Shanghai Jiao Tong University School of Medicine, Shanghai, China

**Keywords:** pituitary adenoma, macrophages, tumor microenvironment, immune cell, therapy

## Abstract

Macrophages are one of the most common infiltrating immune cells and an essential component of tumor microenvironment. Macrophages and the soluble cytokines and chemokines produced play an important role in tumorigenesis, progression, invasion and metastasis in solid tumors. Despite the multiple studies in other solid tumors, there is little known about macrophages in pituitary adenomas. Recently, studies about pituitary adenoma-infiltrated macrophages have been emerging, including the immunohistochemical and immunophenotypic analysis of the pituitary adenomas and further studies into the mechanism of the crosstalk between macrophages and tumor cells *in vivo* and *in vitro*. These studies have offered us new insights into the polarization of macrophages and its role in tumorigenesis, progression and invasion of pituitary adenomas. This review describes the advances in the field of pituitary adenoma-infiltrated macrophages and the prospect of targeting macrophages as cancer therapy in pituitary adenoma.

## 1 Introduction

Pituitary adenoma (PA) is a common brain tumor with a prevalence of 1/865 to 1/2688 according to a study in 2014 (1–3). Neurosurgery is the first-choice treatment for PAs except for prolactinomas, for which dopamine agonists are a preferable treatment. A dopamine agonist normalizes serum prolactin (PRL) and shrinks tumor volume, but 10–30% of cases do not undergo remission with the maximum tolerated dose (4–7); these cases are known as dopamine agonist-resistant prolactinomas (8). PAs are usually slow-growing tumors, while 35–55% present as invasive (9, 10). The invasiveness of PAs increases the difficulty in achieving complete surgical excision, and the postoperative recurrence rate is 46% (11). A small proportion of PAs cannot be cured after conventional treatment and are referred to as refractory pituitary adenomas (12, 13); these are characterized by a rapid growth rate, invasion into surrounding structures, refractory behavior to conventional treatment and severe symptoms (12). Further studies are needed to find more effective therapies for dopamine agonist-resistant prolactinomas, invasive PAs and refractory PAs.

The tumor microenvironment (TME) consists of tumor cells, immune cells, mesenchymal cells, enzymes, growth factors, cytokines and chemokines within the extracellular matrix (ECM) and plays an important role in tumorigenesis, progression and metastasis in solid tumors (14). Tumor-associated macrophages (TAMs) are macrophages that are affected by multiple components and that intervene in the survival, invasiveness and apoptosis of tumor cells through a variety of mechanisms in the TME (15). TAMs commonly express CD68, CD11b and F4/80 and have two origins: bone marrow-derived monocytes in peripheral blood and tissue-resident macrophages of embryonic origin (16). TAMs polarize into two subgroups, M1-TAMs and M2-TAMs, which are subjected to fibrosis, hypoxia, metabolic stress and lymphocyte-secreted factors (17–20). M1-TAMs, which typically express CD80, CD86, MHC II and CD64, usually inhibit tumors through reactive oxygen species (21), antibody-dependent cytotoxicity (22) and NK cell activation (23). In contrast, M2-TAMs, which typically express CD163, CD206 and ARG1, possess a pro-tumoral function (16) through vascularization (24), growth factors (25), ECM degradation (26), immune suppression (27–30) and promotion of epithelial-mesenchymal transition (EMT) (31).

Recently, studies on pituitary adenoma-infiltrating macrophages have emerged, which provide new insights into the polarization and role TAMs play in the invasiveness of PAs. This review describes the advances in the field of macrophages in the pituitary adenoma-tumor microenvironment (PA-TME) and the prospect of targeting macrophages as a therapy for PAs.

## 2 The Infiltration of Macrophages in Human PAs

### 2.1 Macrophage Infiltration in Different Subtypes of PAs

Recent studies have reported CD68+ macrophages in PAs at levels three times higher than those in the normal pituitary (32, 33), and these cells are the most highly infiltrating immune cells in PAs (34). Detected mostly by immunohistochemistry, infiltrating macrophages vary greatly among different PA subtypes. In a study that included 20 nonfunctioning pituitary adenomas (NFPAs) and 50 functional PAs, Zhang et al. reported that the CD68+ macrophage infiltration of growth hormone (GH)-secreting adenomas and prolactin (PRL)-secreting adenomas was significantly higher than that of NFPAs and adrenocorticotropic hormone (ACTH)-secreting adenomas (35). A study by Marques et al., which included 16 NFPAs and 8 GH-secreting adenomas, showed no significant difference in macrophage infiltration between these two adenoma types (34). Principe et al. found that gonadotropin-secreting adenomas (n=12) had higher CD68 expression than other functional PAs (n=16) (33). Another specimen analysis including 44 NFPAs and 28 functional PAs revealed no significant difference in CD68+ cell infiltration between NFPAs and functional PAs, while among functional PAs, PRL-secreting adenomas had higher CD68+ cell infiltration than ACTH- and GH-secreting adenomas (32). Lu’s team, however, performed a more pathologically detailed analysis with 9 densely granulated GH-secreting adenomas, 9 sparsely granulated GH-secreting adenomas, 9 null cell adenomas and 8 ACTH-secreting adenomas. They found that null cell adenomas and sparsely granulated GH-secreting adenomas had higher CD68+ cell infiltration than the other two subtypes (36). For CD163, an M2-TAM marker, few relevant statistical results on its expression in PAs have been published. Using bioinformatics analysis, Yeung et al. reported that NFPAs had higher CD163 expression than functional PAs (37). According to Principe et al., CD163+ cell infiltration of gonadotropin-secreting adenomas was higher than that of other PAs (33).

Based on the current results, no obvious pattern of macrophage infiltration was observed in different subtypes of PAs. A study with a larger sample size supports the conclusion that PRL-secreting adenomas are more highly infiltrated by macrophages, while ACTH-secreting adenomas, gonadotrophin cell adenomas or NFPAs are infiltrated to a lesser extent (35). However, Principe et al. demonstrated that gonadotrophin cell adenomas had higher CD68+ cell infiltration than other functional PAs. Clinically, gonadotrophin cell adenomas are the main pathological subtype of NFPAs. Moreover, they found higher F4/80+ cell infiltration in tumors originating from gonadotrophin cell lines than in tumors originating from GH cell lines in a tumor-bearing animal model. This difference was no longer significant when tumorigenic cells were replaced by human PA primary cells (33). According to the results of this study, macrophage infiltration varied greatly among samples, which indicated that gonadotropin-secreting adenoma has substantial heterogeneity in macrophage infiltration. Several reasons are considered possible. First, Yagnik et al. found that the infiltration of CD11b+ myeloid macrophages in NFPAs differed greatly between samples (38). For null cell adenomas, a subtype of NFPA, it was observed that CD68+ macrophage infiltration was higher than that in ACTH-secreting adenomas and densely granulated GH-secreting adenomas (36), which indicates that null cell adenomas may have affected the statistical results of NFPAs in other studies. Although null cell adenomas only account for a small proportion of NFPAs, they should be studied separately from the other pathological types of NFPAs in future studies. Second, chemokines might be expressed differently among subtypes of PAs. CCL5 was found to be expressed higher in GH adenomas than in gonadotrophin adenomas, while CSF1 was found to be expressed more in gonadotrophin adenomas. Meanwhile, gonadotrophin-secreting cell lines could increase the expression of CSF1R on monocyte cell line THP1, compared to GH cell lines (33). Marques et al. have similar results: they found that NFPAs could secret more IL-8, CCL2 and CCL4 than GH adenomas (34). Due to the differential results shown above, macrophages infiltration must be the consequence of the secretion of multiple chemokines. Thus, the marker of macrophage subgroup in different pituitary adenomas should be further studied. Third, differential tumor development stage with differential chemokines expression could lead to differential macrophage infiltration. Yagnik et al. inferred a dynamic change of macrophages infiltration in the development of NFPAs, caused by dynamic chemokines expression, such as GM-CSF and MCP-1, supporting the opinion mentioned above (38).

### 2.2 The Relationship Between Macrophages and the Biological Behavior of PAs

Multiple studies have reported a correlation between TAMs and the biological behavior of PAs, such as tumor growth and invasion. Regarding PA growth indicators, after studying PA specimens, Lu et al. and Zhang et al. found that CD68+ macrophage infiltration was related to the size of PAs (35, 36). Principe et al. found that the percentage of CD68+ cells was positively correlated with tumor size in gonadotropin cell adenomas (33). In terms of PA invasion indicators, Lu et al. team found that CD68+ macrophage infiltration was positively correlated with the Knosp classification grade, and sparsely granulated GH adenomas, which are considered to be more aggressive, had more CD68+ macrophage infiltration than densely granulated GH-secreting adenomas (36). An analysis by Zhang et al. showed that CD68+ macrophage infiltration was significantly increased in invasive PAs compared with noninvasive PAs (35). Principe et al. reported that CD68+ macrophage infiltration was related to invasion in both functional PAs and NFPAs (33). Yagnik et al.’s found that the M2/M1 gene expression ratios of 88% NFPA samples with cavernous sinus invasion was higher than one (38). These results show that macrophage infiltration plays an important role in tumor growth and invasion. And among the two subtypes of TAMs, M2-TAMs seem to have a positive relation with PA invasion compared to M1-TAMs.

In addition to tumor growth and invasion, the PA-related biological behaviors of clinical concern include drug resistance, recurrence, and even metastasis. However, no convincing data support the relationship of these behaviors with macrophage infiltration. Different opinions also exist about the relationship between PAs and macrophage infiltration. As mentioned earlier, a recent analysis of a larger sample size of pathological specimens found that macrophage infiltration is related to the growth and invasion of PAs (33, 35, 36). However, according to the study by Marques et al., CD68+ macrophage infiltration was not involved with cavernous sinus invasion and was not related to the Ki67 index, which might be attributed to the study’s relatively small sample size (34).

## 3 Crosstalk Between Macrophages and Tumor Cells in the PA-TME

Similar to what is observed in other solid tumors, macrophages in the PA-TME promote the progression of PA cells through multiple mechanisms. Moreover, macrophages in PAs are also regulated by various components in the TME, which allow them to polarize into M2-TAMs and exhibit PA-promoting phenotypic and functional characteristics. Advances in the knowledge of the interaction and regulation of macrophages and TME components in PA are described below.

### 3.1 The Effect of M1- and M2-TAMs on Tumor Cells in the PA-TME

Several studies have shown that M2-TAMs are the primary infiltrative macrophage subtype (33, 34, 37). However, Yagnik et al. also observed that a few specimens were predominantly infiltrated with M1-TAMs rather than M2-TAMs (38). M1-TAMs, which are traditionally considered an anti-tumoral cell type, may promote immunity and suppress tumor growth in PAs. In 1993, TtT/M-87 was separated from PAs. TtT/M-87 is a macrophage cell line derived from thyroid stimulating hormone (TSH)-secreting adenomas that express TNF-α, IL-1α, and MHC-II; these factors can assist spleen-derived lymphocytes in inhibiting the growth of tumor cell lines *in vitro* (39). Due to the lack of relevant research, further *in vivo* and *in vitro* studies are needed to verify the role of M1-TAMs in PAs.

**Figure 1:**
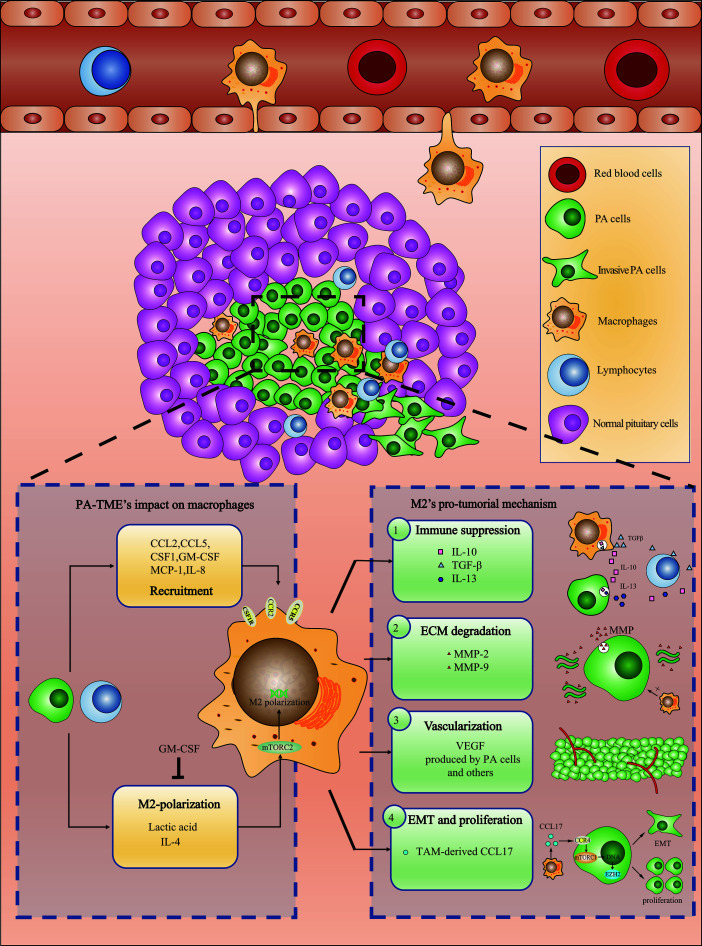
Recruited macrophages polarize into M2-TAMs and promote PAs invasion through a variety of mechanisms.

M2-TAMs promote PA invasion by immunity suppression, PA cell epithelial-mesenchymal transition and proliferation, vascularization and ECM remodeling (40) (**Figure 1**), as described below.

#### 3.1.1 Immunity Suppression

A study of 72 PA specimens found that CD163 expression in tumors is related to inhibitory molecules, such as PD-L1, PD-L2, and LAG3 (32). Treatment with PA cell conditioned medium (CM) upregulated the expression of IL-10 and TGF-β in THP-1 cells, a monocytic cell line (35). Similarly, CM from a macrophage cell line could also upregulate the expression of IL-10, IL-13 and other cytokines in the PA cell line GH3 (34). From these results, M2-TAMs are responsible for immunosuppressive microenvironment in PA.

#### 3.1.2 Epithelial-Mesenchymal Transition and Proliferation

Studies have reported that macrophages can promote EMT in PA cells and increase their invasiveness. When a PA cell line was treated with macrophage-derived CM, its cell morphology changed. At the same time, ZEB-1, a mesenchymal marker, was upregulated, while the epithelial marker E-cadherin was downregulated (34, 41). This may be related to the effect of CCL17 secreted by TAMs (35). CCL17 interacts with CCR4 on PA cells, activates the mTORC1 pathway, and ultimately leads to EMT, which promotes invasion and proliferation (35). When tumor cells undergo EMT, they gradually abandon their epithelial features and turn into a mesenchymal form, during which their ability of invasion and metastasis is promoted. In PAs, EMT marker was found to be associated with tumor size and staging (42). Yagnik et al. found that CM of M2-TAMs could upregulate the expression of EZH2 in NFPA primary cells (38). EZH2 is a proliferation-related gene that, when silenced, abrogated the pro-tumoral effect of macrophage-derived CM. In PAs, EZH2 is correlated with Ki67, and therefore possibly related to the proliferation of PA cells (43). Therefore, EZH2 expression could be the consequence of CCL17-induced EMT transcription, leading to proliferation, which still needs further validation.

#### 3.1.3 Vascularization

Many studies have found that M2-TAMs in PAs are positively correlated with microvessel density and VEGF expression (34, 44, 45). Marques reported that the chemokine CCL2, which recruits macrophages, was significantly correlated with the microvessel area of PA specimens, as was the ratio of M2-TAMs to M1-TAMs (45). Yagnik et al. found that CM of M2-TAMs could upregulate the expression of S100A9 in NFPA primary cells (38). S100A9 is an invasion-regulating protein that, when silenced, can inhibit the invasion and migration of primary NFPA cells induced by macrophage-derived CM (38). In addition, S100 protein was found to be associated with VEGF and EGFR expression, which is closely related to vascularization and invasion (46). Based on the fact that folliculo-stellate cells and tumor cells can produce VEGF (47) and that PA cells can express S100A9, vascularization could be the consequence of VEGF derived from cells mentioned above and aggregation of TAMs could just be the result of vascularization. Therefore, the role TAMs play is still in need of further validation and mechanism researches.

#### 3.1.4 ECM Remodeling

CD301 and ARG1, which are both M2-TAM markers, are positively correlated with the expression of the matrix metalloproteinases MMP-2 and MMP-9 in PA specimens, while M1 markers have no such relationship (35). MMP-2 and MMP-9 expression was found to be more abundant in invasive pituitary adenoma (48). Macrophage-derived CM can upregulate the transcription of MMP-9 mRNA in PA cell lines ***in vitro*** (34). As proteases, MMPs degrade the ECM and promote tumor invasiveness, which has been reported in a variety of solid tumors (49, 50). However, it was reported folliculo-stellate cells could secret MMP-9 and degrade ECM (51). Therefore, based on current results, TAM might upregulate the expression of MMPs and degrade ECM indirectly.

### 3.2 The Effect of the PA-TME on TAM Polarization

Studies have found that metabolites, cytokines, chemokines and other factors can act on macrophages, affect their phenotypes, and promote their polarization to M2-TAMs (52). In PAs, little is known about factors that affect TAM polarization. Through *in vivo* and *in vitro* models, Zhang et al. demonstrated that lactic acid could activate the mTORC2 pathway to cause M2-type polarization and promote the expression of multiple factors in macrophages, such as CCL17, CCL22, IL-1α, IL-10, and TGF-β (35). Marques et al. found that the IL-4 level was 5 times higher than that of IFN-γ in the PA-TME (34). Both IL-4 and IFN-γ are well-known cytokines that polarize macrophages to M1 and M2 types, respectively. Lactic acid is due to the anaerobic environment inside the solid tumor. The source of IL-4 and IFN-γ could be tumor cells, the tumor-infiltrating lymphocytes, mesenchymal cells or macrophages. A recent study found that in lung cancer, IL-4 secreted by M2-myeloid cells and tumor cells activate the STAT6 pathway and promote M2 polarization and tumor progress (53). Similarly, PA cells and TAMs might be the potential source of IL-4 and worth exploring. During the development of PAs, the balance of M1 and M2 macrophages changes based on factors in the TME. Yagnik et al. found that as the number of CD11b+ myeloid cells increased, the expression of the M1-TAM marker CD64 increased. Compared with CM from M1-TAMs, CM from M2 macrophages reduced the expression of MCP-1 in NFPA cells. They also observed that GM-CSF inhibition abrogated the M1 polarization of THP-1 cells induced by CM from NFPA cells. These results suggested dynamic changes that affect the balance of M1- and M2-TAM infiltration in NFPA. Therefore, they divided NFPA into M1-NFPA and M2-NFPA according to the percentage of M1- and M2-TAM infiltration. Assuming the tumors behaved as M2-NFPA during early formation, infiltrating monocytes differentiated into M2-TAMs due to MCP-1 produced by tumor cells. However, over time, the aggregated M2-TAMs downregulated MCP-1 and upregulated GM-CSF expression in PA cells. As suggested above, GM-CSF can induce the differentiation of monocytes into M1-TAMs, while the MCP-1 downregulation can also slow monocyte recruitment. Therefore, as time passed, the proportion of infiltrating M1-TAMs gradually increased (38). Fujawara et al. found that the number of infiltrating macrophages increased even before any tumors formed and that they were mainly M2 macrophages, with few M1 macrophages (54). Such results supported Yagnik’s theory, which at least partially explains the heterogeneity of macrophage infiltration in NFPAs. NFPAs intrinsically have different statuses of macrophage infiltration, which has caused substantial differences among the results in the PA studies mentioned above. This theory has provided a potential transition for PAs from M2-dominant to M1-dominant status. Inferred from their theory, most PAs must end up in an M1-dominant status. However, according to Yeung et al.’s results of 134 patients, infiltration of M2-TAMs is far more than that of M1-TAMs in all subtypes of PAs. Therefore, uncovered molecular feedback could exist to shift the balance to M2-type and needs exploring.

## 4 Future Prospects

Regarding the clinical dilemma in the treatment of pituitary adenomas, PA invasion exhibits the strongest relationship with macrophages. Invasive PAs can invade the parasellar structure, which causes severe symptoms, incomplete surgical resection and recurrence. Many studies have found that macrophage infiltration is related to invasion and progression indicators, including tumor size and Knosp classification grades (33, 35, 36). Therefore, further research on macrophages is necessary to solve the problem of PA invasion. Drug resistance and recurrence are two other major problems in PAs that need to be solved, but no macrophage-related research has been published on these topics. Studies in these two areas will further enhance the practicality of macrophage-related research in PAs.

### 4.1 Exploration of the Infiltration Patterns of Macrophages in Different Subtypes of PA

Although most studies support the relationship between macrophages and PAs, the relationship may be complicated. A recent study analyzed 140 PAs and 20 normal pituitary tissues and found that PAs can be divided into three subtypes according to immune cell infiltration, of which only one subtype is characterized by relatively higher levels of infiltrating macrophages (55). This indicates that the role of macrophages in PA may not be simply summarized as purely “relevant” or “irrelevant”.

As mentioned above, the pattern of macrophage infiltration among different subtypes of PAs has not been established. The infiltration of CD68+ cells and CD163+ cells in NFPAs and functional PAs varies among different studies (32, 33, 35). NFPAs include tumors with a variety of pathological classifications. Clinically, NFPAs are mainly composed of gonadotropin cell adenomas. In addition, this tumor subtype also encompasses null cell adenomas and hormone-silent adenomas (56). Marques et al. reported on different subtypes of NFPAs and found no difference in macrophage infiltration among gonadotropin cell adenomas, silent ACTH adenomas and null cell adenomas. However, only 1–2 cases of the latter two were observed, which was not enough for a convincing conclusion (34). Therefore, it is not advisable to study relevant mechanisms and therapies without knowing the pattern of macrophage infiltration of different PA subtypes. Large multicenter studies are needed to clarify the pattern mentioned above and to further clarify the relationship between macrophages and PA invasion, drug resistance and recurrence.

Several directions are considered promising. Barry et al. found that AIP-mutated tumors showed an increased infiltration of CD68+ macrophages comparing to sporadic GH tumors (41). Thus, AIP-related pathway should be paid attention to. A study had found a correlation between AIP and cAMP signaling (57). Thus, the activation of PKA/cAMP pathway might explain the heterogeneity mentioned above. In addition to that, Principe et al.’s study found that the infiltration of CD4+T and CD8+T rather than NK cells and B cells was higher in functional PAs than gonadotropin-secreting adenomas, which was the main component of NFPAs (33). Zhou et al. ran RNA-seq on 115 human PAs and found B cells, CD8+ T cells and CD4+ T cells were negatively correlated, while NK cells were positively correlated with macrophage infiltration. The infiltration of T cells and B cells in functional PA was significantly higher than that of NFPA, especially in GH-secreting adenoma. Meanwhile, they found that higher CD8+T and CD4+T infiltration corresponded to stronger tumor invasiveness and worse survival (58). Mei et al.’s results also suggested functional PAs had a higher CD4+T and CD8+T infiltration than NFPAs (32). From these results, functional PAs seem to attract more CD4+T and CD8+T cells than NFPAs. Considering the negative relation between these lymphocytes and macrophages, T cells might affect the recruitment of macrophages. The molecular and genetic mechanism underneath needs to be further studied.

### 4.2 Further Exploration of the Origin of TAMs in PAs

Traditionally, TAMs are believed to enter tumors as a result of chemotaxis of peripheral blood mononuclear cells. However, studies have reported embryonic macrophages in the TME of lung cancer and glioma (59, 60). At present, studies on the origin of PA-TAMs are still in the initial stages. Yagnik et al. found that CD11b+ myeloid cells in NFPAs expressed CCR5 rather than CX3CR1. CCR5 is expressed by recruited macrophages, while CX3CR1 is expressed by microglia, which are tissue-resident macrophages in the brain (38). However, Yagnik et al.’s research was limited to one PA subtype, and whether this was a common phenomenon in other subtypes remains to be determined.

### 4.3 Exploration of a Novel TAM Pro-Tumoral Mechanism

In recent years, novel pro-tumoral mechanisms of TAMs have been consistently discovered in brain tumors. In gliomas, M2-TAMs can induce CECR1, activate the MAPK pathway, and stimulate the proliferation and migration of tumor cells (61). Another study showed that microglia can induce the expression of PDGFRB in glioma cells, thereby enhancing their migration ability (62). In PAs, little is known about the pro-tumoral mechanism of TAMs. Except for Zhang et al.’s work (35), no other relevant studies have been published. The prospects of mechanistic studies of PA-TAM are described next.

#### 4.3.1 Exploring the Crosstalk Between TAMs and PA Cells

Exploring the direct or indirect effect of TAMs on PA cells is the most obvious direction to further study the pro-tumoral mechanism of TAMs. Moreover, TAMs are directly and indirectly affected by PAs, and their phenotype and function undergo a series of changes. Recently, a study reported that exosome released from tumor cells, which contained KRAS protein, drive TAM polarization, thus promoting tumor growth. Whether such interaction between TAMs and PA cells exists is worth exploring (63).

#### 4.3.2 Exploration of the Role of Metabolism in the Pro-Tumoral Mechanism of M2-TAMs

The TME is considered a harsh environment that is acidic, hypoxic, and lacks nutrients (64). Low sugar and lipid levels can cause metabolic stress in tumor cells and can lead to the activation of several signaling pathways, such as ROS signaling, AMPK and AKT pathway (65). Whether PA-TAMs can undergo such changes and whether these changes affect the function of PA-TAMs require additional discussion.

#### 4.3.3 Exploration of the Crosstalk Between TAMs and Other Immune cells in the PA-TME

In addition to macrophages, other immune cells are present in the TME, such as CD4+ T cells, CD8+ T cells, B cells, nature killer cells, dendritic cells, Tregs and myeloid-derived suppressor cells (55). The effect of TAMs on PA cells is largely affected by these immune cells. According to Zhou et al., CD8+ T cells and CD4+ T cells were negatively correlated, while NK cells were positively correlated with macrophage infiltration. Meanwhile, they found that higher CD8+T and CD4+T infiltration corresponded to higher PD-1/PD-L1 expression, stronger tumor invasiveness and worse survival (58). Their findings suggested a immunosuppressive microenvironment in PAs, probably caused by suppressive factors released by lymphocytes. PAs infiltrated T cells could react on M1-TAMs and hinder their anti-tumoral functions, thus leading to immune suppression. Therefore, the crosstalk between PA-TAMs and other immune cells and how this interaction promotes PA progression are valuable research directions.

#### 4.3.4 Interactions Between Macrophages and Folliculo-Stellate Cells

Folliculo-stellate cells are non-endocrine cells in the anterior pituitary, and their existence has been reported in PAs (66). In the normal pituitary, folliculo-stellate cells can secrete factors, such as TGF-β1, TGF-β3, bFGF and IL-6 (67). One study of 286 GH-secreting adenomas found that 69% of the tumors contained folliculo-stellate cells. Those authors also found that follicular stellate cells in PAs might be related to preoperative serum GH levels (68). In the PA-TME, whether follicular stellate cells affect M1- or M2-TAMs is still unclear, but their relationship is worth exploring.

#### 4.3.5 Interaction Between Macrophages and Fibroblasts

Clinically, PA stiffness is an important factor that affects the operative approach and the success of surgery. Collagen produced by fibroblasts is an important factor that affects tumor stiffness (69). Study on the interaction between macrophages and fibroblasts and how TAMs can impact the collagen produced in the PA-TME may provide ideas for solving the problem of PA stiffness.

### 4.4 Research Prospects of TAM-Targeted Therapy in PA

Studies on macrophage-targeted therapy have been widely performed in other tumors (70). In solid tumors, the mechanisms of macrophage-targeted therapy mainly include macrophage depletion, macrophage recruitment blockade and macrophage reprogramming. The classification according to the mechanism and the relevant information of the corresponding representative drugs are shown in **Table 1**. The CSF1-CSF1R axis is necessary for macrophage differentiation and survival (78). Targeting CSF1R with a CSF1R monoclonal antibody or small molecule inhibitor can effectively deplete macrophages (79). Principe et al. found that blocking CSF1 can inhibit the migration of THP1 monocytes toward PA cells *in vitro* (33). This indicates that targeting the CSF1-CSF1R axis in PA may not only deplete macrophages but also reduce their recruitment. Targeting macrophage recruitment is another strategy for macrophage-targeted therapy. This strategy primarily targets chemokine pathways using CCL2 and CCR2 monoclonal antibodies to reduce macrophage recruitment. A study found that in esophageal cancer, blocking the CCL2-CCR2 axis could inhibit the recruitment of TAMs, thereby enhancing the anti-tumor immunity of CD8+ T cells in the TME (80). In PAs, it was found that CCL2 expression was higher than in the normal pituitary (34). Barry et al. found that blocking CCR5 could inhibit the migration of macrophages induced by CM of the GH3 cell line (41). Zhang et al. reported that the CCR4 antagonist AZD2098 significantly inhibited the promotive effect of TAM-derived CCL17 on the proliferation, invasion and migration of the PA cell line GH3 *in vitro* and reduced tumor burden in GH3 tumor-bearing mice *in vivo* (35). Therefore, targeting CCL2, CCR4 or CCR5 may also reduce the recruitment of macrophages to the PA-TME. Using multiple methods, M2-TAMs can be reprogrammed into M1-TAMs with anti-tumor properties. In other solid tumors, macrophage reprogramming therapy includes the use of anti-CD47 antibodies, anti-CD40 antibodies, Toll-like receptor (TLR) agonists, histone deacetylase (HDAC) inhibitors and PI3K inhibitors (76, 77, 81, 82). In PAs, Zhang et al. found that lactic acid induced M2 polarization of TAMs through the mTORC2 pathway. Additionally, CM from GH3 cells treated with an LDHA inhibitor significantly reduced M2 markers in THP-1 cells compared with the control group (35). Therefore, targeting LDHA or mTORC2 may repolarize M2-TAMs to M1-TAMs. A few reviews have recently summarized the drugs that target macrophages in cancer (83, 84). These drugs will allow for PA macrophage-targeted therapy in the future. Related drugs and possible targets in PAs are shown in **Table 1**.

**Table 1:** The mechanisms, corresponding drugs, the potential targets in PAs and relevant information of macrophage targeted therapy.

Mechanism	Targets in other solid tumors	References	Drug name	Drug category	Solid tumors	Phase	NCT number	Potential targets of PA-TAM	Research progress of PA-TAM potential targets	References of PA-TAM potential targets
Macrophage depletion	CSF1R	2017, Yan (71)	PLX3397	CSF1R inhibitor	Giant cell tumors of the tendon sheath	III	NCT02371369	CSF1R	*In vitro* experiment	2020, Principe (33)
					Breast cancer	Ib/II	NCT01596751			
			BLZ945		Advanced solid tumor	I/II	NCT02829723			
			ARRY-382		Advanced solid tumor	II	NCT02880371			
			JNJ-40346527		Prostate cancer	I	NCT03177460			
			FPA008	CSF1R monoclonal antibody	Tenosynovial giant cell tumor	I/II	NCT02471716			
			IMC-CS4		Advanced solid tumor	I	NCT01346358			
			RG7155		Breast cancer, Ovarian cancer	I	NCT02323191			
Macrophage recruitment blockade	CCL2-CCR2	2016, Fang (72)	CNTO 888	CCL2 monoclonal antibody	Prostate cancer	II	NCT00992186	CCL2-CCR2,	/	2019, Marques (34)
			MLN1202		Metastatic cancer	II	NCT01015560	CCR4	*In vitro* and *in vivo* experiments	2021, Zhang (35)
								CCR5	*In vitro* experiment	2019, Barry (41)
Macrophage reprogramming	CD47	2010, Chao (73)	Hu5F9-G4	CD47 monoclonal antibody	Colorectal cancer	I/II	NCT02953782	LDHA	*In vitro* experiment	2021, Zhang (35)
			TTI-621	SIRP1α-Fc fusion protein	Small Cell Lung Cancer	I	NCT02663518			
	TLR	2013, Le Mercier (74)	IMO-2125	TLR7 ligand	Melanoma	III	NCT03445533			
			CMP-001	TLR9 ligand	Melanoma	II	NCT03618641			
			SD101		Solid tumor	II	NCT03007732			
	CD40	2018, Perry (75)	APX005M	CD40 monoclonal antibody (agonist)	NSCLC	I/II	NCT03123783	mTORC	/	
			R07009879		Advanced solid tumor	I	NCT02760797			
			SEA-CD40		Solid tumor	I	NCT02376699			
			CP-870,893		Melanoma	I	NCT01103635			
	HDAC	2017, Guerriero (76)	Vorinostat	HDAC inhibitor	Multiple myeloma	III	NCT00773747			
	PI3K	2016, Megan (77)	BAY80-6946	PI3K inhibitor	lymphoma	III	NCT02626455			

## 5 Conclusion

Generally, research on macrophages in the PA-TME is relatively scarce, and many factors are considered responsible. First, the sample sizes of previous PA studies were small and should be further expanded. Second, it is also difficult to immortalize primary cells from PAs and to maintain stable passage and hormone expression (85). Finally, few research teams exist worldwide, and funding is limited. These reasons have caused the current dearth of PA-TAM research.

Macrophages in PAs can interact with tumor cells, mesenchymal cells, soluble factors and other TME components to affect the invasiveness, drug resistance and recurrence of PAs. There is great potential for the prospect of PA-TAMs. Further research may provide new treatments for PAs and provide new approaches to overcome the current predicament of PAs.

## Author Contributions

Conception and design of the review: ZW and SL. Drafting the manuscript and the figure: CH. Modifying the manuscript critically for important content: SL, XL and LX. All authors contributed to the article and approved the submitted version.

## Funding

This work was supported by the National Natural Science Foundation of China (grant Nos. 81671371 and 81972339 to ZBW and 81701359 to SL).

## Conflict of Interest

The authors declare that the research was conducted in the absence of any commercial or financial relationships that could be construed as a potential conflict of interest.

## Publisher’s Note

All claims expressed in this article are solely those of the authors and do not necessarily represent those of their affiliated organizations, or those of the publisher, the editors and the reviewers. Any product that may be evaluated in this article, or claim that may be made by its manufacturer, is not guaranteed or endorsed by the publisher.
